# Textural features of the frontal white matter could be used to discriminate amnestic mild cognitive impairment patients from the normal population

**DOI:** 10.1002/brb3.3222

**Published:** 2023-08-17

**Authors:** Wei Zheng, Ronghua Mu, Fuzhen Liu, Xiaoyan Qin, Xin Li, Peng Yang, Xin Li, Yahui Liang, Xiqi Zhu

**Affiliations:** ^1^ Department of Clinical Medicine Guilin Medical university Guilin China; ^2^ Department of Medical Imaging Nanxishan Hospital of Guangxi Zhuang Autonomous Region Guilin China; ^3^ Guilin Medical University Guilin China

**Keywords:** amnestic mild cognitive impairment, frontal lobe, magnetic resonance imaging, radiomics, white matter

## Abstract

**Objective:**

We aim to develop a radiomics model based on 3‐dimensional (3D)‐T1WI images to discriminate amnestic mild cognitive impairment (aMCI) patients from the normal population by measuring changes in frontal white matter.

**Methods:**

In this study, 126 patients with aMCI and 174 normal controls (NC) were recruited from the local community. All subjects underwent routine magnetic resonance imaging examination (including 3D‐T1WI ). Participants were randomly divided into a training set (*n* = 242, aMCI:102, NC:140) and a testing set (*n* = 58, aMCI:24, NC:34). Texture features of the frontal lobe were extracted from 3D‐T1WI images. The least absolute shrinkage and selection operator (LASSO) was used to reduce feature dimensions and develop a radiomics signature model. Diagnostic performance was assessed in the training and testing sets using the receiver operating characteristic (ROC) curve analysis. The area under the ROC curve (AUC), sensitivity, and specificity were also calculated. The efficacy of the radiomics model in discriminating aMCI patients from the normal population was assessed by decision curve analysis (DCA).

**Results:**

A total of 108 frontal lobe texture features were extracted from 3D‐T1WI images. LASSO selected 58 radiomic features for the final model, including log‐sigma (*n* = 18), original (*n* = 8), and wavelet (*n* = 32) features. The performance of radiomic features extracted from 3D T1 imaging for distinguishing aMCI patients from controls was: in the training set, AUC was 1.00, and the accuracy, sensitivity, and specificity were 100%, 98%, and 100%, respectively. In the testing set, AUC was 0.82 (95% CI:0.69–0.95), and the accuracy, sensitivity, and specificity were 69%, 92%, and 55%, respectively. The DCA demonstrated that the model had favorable clinical predictive value.

**Conclusions:**

Textural features of white matter in the frontal lobe showed potential for distinguishing aMCI from the normal population, which could be a surrogate protocol to aid aMCI screening in clinical setting.

## Graphical abstract

(a) Drawing regions of interest in frontal lobe using ITK‐SNAP. (b) Strategy for frontal lobe extraction from high‐resolution structural magnetic resonance imaging. (c) Intensity features and textural features were extracted from the images, and used the least absolute shrinkage and selection operator to choose the optimized subset of features to construct the final model. (d) Statistical analysis was used to find radiomic features. aMCI, amnestic mild cognitive impairment.

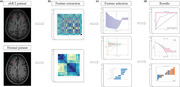

## 1 INTRODUCTION

Mild cognitive impairment (MCI) is a transitional state between normal aging and dementia disorders, especially Alzheimer's disease (AD; Petersen et al., 1999), which means an individual representing abnormal cognitive decline while keeping functional independence (Petersen et al., 1999). However, there is currently no effective clinical strategy to prevent or cure AD (Gauthier et al., 2006). Therefore, the hotpot of the current research has gradually shifted to study MCI. Retrospective studies estimate prevalence of MCI ranged from/span > 16% to 20% (Roberts & Knopman, 2013). According to the current clinical criteria, MCI patients identified could be classified into one of the two categories: amnestic MCI (aMCI) and nonamnestic MCI (naMCI; Petersen et al., 2014). With the development of the disease, aMCI might progress to AD, and naMCI might progress to non‐AD dementias (Roberts & Knopman, 2013). A systematic review found that an average of 32% of patients with MCI developed into AD within 5 years, indicating that MCI patients have a high risk of conversion to AD (Alzheimer's & Dementia, 2022). Therefore, the identification and prevention of aMCI is of great importance.

MCI differs from dementia in many ways, and previous studies support better outcomes for early treatment or prevention of MCI (Pilipovich & Vorob'eva, 2020). Biomarkers like cerebrospinal fluid concentrations of amyloid beta (1‐42), phospho‐tau, and total tau protein estimated had been established for the identification of MCI (Kasper et al., 2020). Although these cerebrospinal fluid markers perform well enough to have a role in the clinical work‐up of patients with dementia (Kasper et al., 2020), this invasive examination limits the application in the clinical setting for MCI screening. Developing a new noninvasive modality to effectively identify the MCI is urgently needed.

Radiomics, as a rapidly advanced image analysis technique, has gained increasing attention in recent years. Radiomics extracts the high‐throughput features from segmented regions and quantitatively analyzes the lesion heterogeneity using appropriate models (Kumar et al., 2012). Previous study found on radiomics models based on structural images of the hippocampus had a good diagnostic performance for AD discrimination (Wang et al., 2022). The essentially pathological signs of AD might interfere with the integrity of white matter because of extracellular amyloid‐b protein deposits and intracellular neurofibrillary tangles at the molecular level, which often occur together with structural changes resulting in atrophy of cortical and subcortical structures (Defrancesco et al., 2014). Those structural changes of the brain reflect an early sign of AD onset in MCI patients and consequently lead to MCI learning and memory deficits (Defrancesco et al., 2014). In evidence, postmortem results showed that the pathological features of aMCI were consistent with those of very early‐stage AD (Petersen et al., 2006). Furthermore, previous studies using protocols of multi‐parameter magnetic resonance imaging (MRI) have validated that the frontal lobe, specifically in frontal‐subcortical circuits and the integrity of the white matter of the brain, has been damaged in patients of aMCI and AD (Toshkhujaev et al., 2020; Turriziani et al., 2012; Zamani et al., 2022). In addition, repetitive transcranial magnetic stimulation in frontal lobe could improve language and memory function in MCI patients (Turriziani et al., 2012). Therefore, we hypothesized that frontal lobe micro‐structure changes could be radiomics markers of aMCI. In the present study, we conducted a radiomics based on 3D‐T1 MRI images to discriminate aMCI patients from health subjects.

## 2 MATERIALS AND METHODS

This prospective study was approved by the local ethics committee, all recruited participants have signed informed consent.

### 2.1 Subjects

From October 2020 to February 2021, 146 patients with aMCI and 189 healthy volunteers were recruited, all subjects were distributed a label that did not identify the group assignment. The gender, age, education, systolic pressure, and diastolic pressure of all subjects were collected for both groups. Body mass index (BMI, kg/m^2^) was calculated from height and weight, BMI = weight/height^2^. All aMCI patients were verified and recruited by an experienced neurological physician according to the The Diagnostic and Statistical Manual of Mental Disorders‐IV(DSM‐IV) and Petersen diagnostic criteria (Lu et al., 2011; Tang, 2020). We used the Montreal Cognitive Assessment (MoCA) to replace the Mini‐Mental State Examination in evaluating cognitive function. MCI diagnostic criteria are as follows: The MoCA scores of aMCI patients were ≤13 in the illiteracy group, ≤19 in the elementary school group, and ≤24 in the middle school and above group (Lu et al., 2011). The inclusion criteria for the normal control group were as follows: (Petersen et al., 1999) absence of neurological impairment, such as visual loss or hearing loss and (Gauthier et al., 2006) MoCA score ≥ 28. The basic demographic and neuropsychological data are presented.

### 2.2 MRI data acquisition

All MRI protocols were performed using a 3.0T Magnetic Resonance (MR) scanner (Ingenia CX, Philips Healthcare) with a 32‐channel head coil. Scan sequences were listed below: three‐dimensional(3D) T1 fast field echo, repeat time (TR) = 6.4 ms, echo time (TE) = 3.0 ms, field of view (FOV) = 240 × 240 × 180 mm, reconstruction voxel size = 1.1 × 1.1 × 1.1, reconstruction matrix = 400 × 400, slice thickness = 1.1 mm; 3D T2 spin echo, TR = 2500 ms, TE = 232 ms, FOV = 250 × 25 × 180 mm, reconstruction voxel size = 1.1 × 1.1 × 1.1, reconstruction matrix = 512 × 512, slice thickness = 1.1 mm; 3D fluid attenuated inversion recovery, TR = 4800 ms, TE = 244 ms, FOV = 240 × 240 × 173 mm, reconstruction voxel size = 1.1 × 1.1 × 1.1, reconstruction matrix = 384 × 384, and slice thickness = 1.2 mm. Each subject underwent the above MRI sequences.

### 2.3 (ROI) delineated

**FIGURE 1:**
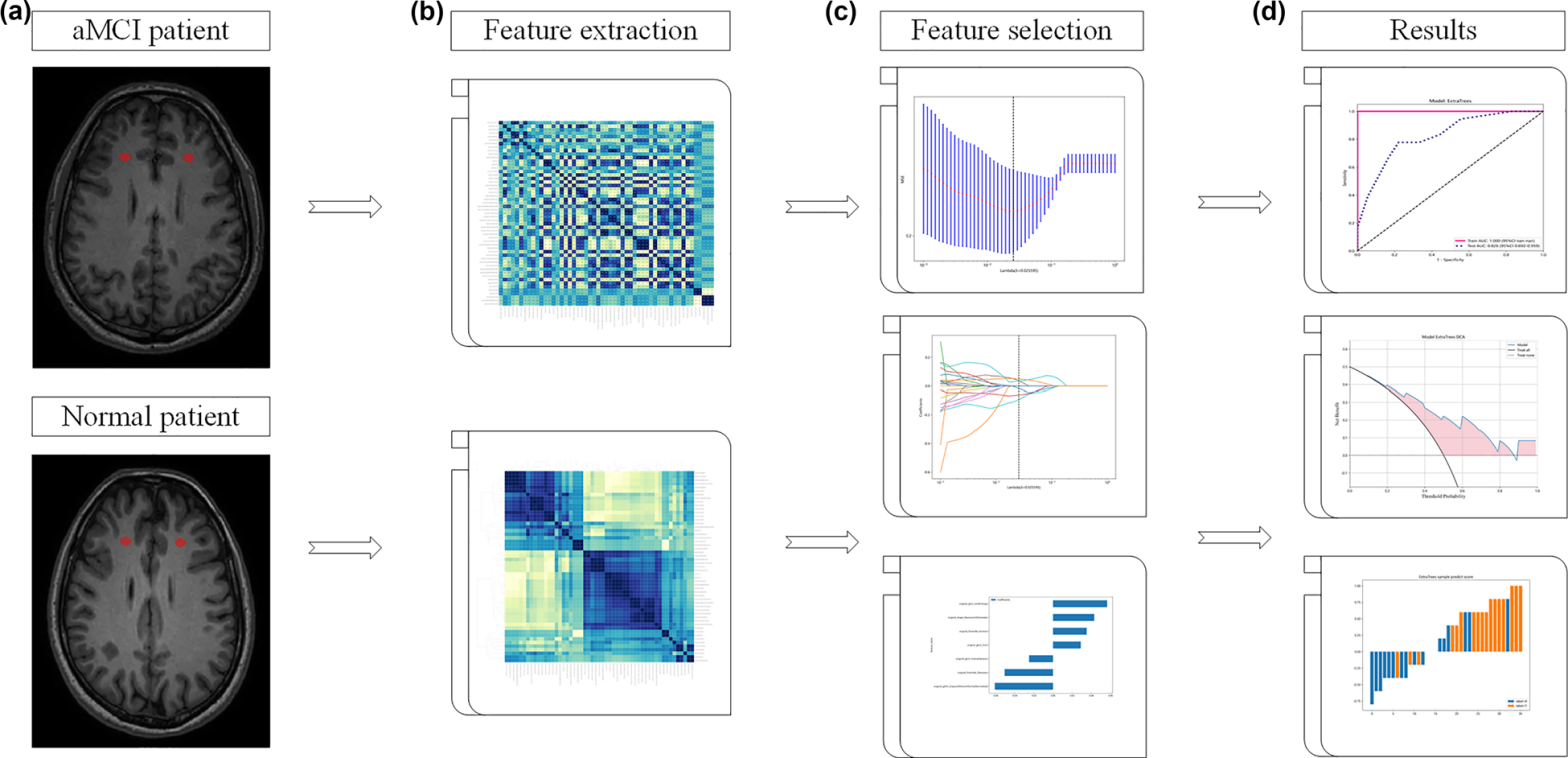
Wikodeling for aMCI patients. (a) Drawing ROIs in frontal lobe using ITK‐SNAP. (b) Strategy for frontal lobe extraction from high‐resolution structural MRI. (c) Intensity features and textural features were extracted from the images, and used the LASSO to choose the optimized subset of features to construct the final model. (d) Statistical analysis was used to find radiomic features. aMCI, amnestic mild cognitive impairment; LASSO, least absolute shrinkage and selection operator; MRI, magnetic resonance imaging; ROIs, regions of interest.

The ROIs were manually delineated on the horizontal T1 images for segmentation using the ITK‐SNAP software (Version 3.6.0, www.itksnap.org). The frontal lobes were drawn manually by a neuroradiologist with 4 years of experience. All ROI results were corrected by another neuroradiologist with 15 years of MRI experience. They were both blinded to the results of clinical and neuropsychological materials (Figure 1).

ROI delineation criteria are as follows: (i) the circular measurement tool was used to delineate the ten consecutive layers in the white matter of frontal lobe; (ii) the size of the bilateral ROI of all subjects remained the same, with an area of about 10 mm^2^; and (iii) areas of abnormal signals shown on MR images were avoided.

One of the above‐mentioned radiologists randomly selected and drew 50 cases independently. After 1 month, radiologist A repeated the same procedure again. An intra‐class correlation coefficient (ICC) greater than 0.75 is considered to indicate good consistency. In our study, we obtained a good ICC result (see Results section for details).

### 2.4 Radiomic features extraction

In this study, radiomic features were extracted using pyradiomics, and 108 features were obtained from 3D MRI T1‐weighted images. Before feature selection, each feature value for all frontal lobes was normalized with Z‐scores ((*x*−*μ)/σ*) (*x* refers to the value of the feature, *μ* represents the average value of this feature for all patients in the cohort, and *σ* indicates the corresponding standard deviation) in order to remove the unit limits of each feature before applying the machine learning model for classification (Figure 1). And all frontal lobes were randomly divided into the training and test sets in a proportion of 8:2.

### 2.5 Features selection

Due to the high complexity of the extracted features, there was a risk of overfitting in the analysis. Hence, the dimensions presented by these quantitative features needed to be reduced by prioritizing the features. The least absolute shrinkage and selection operator (LASSO) is a popular high‐dimensional data analysis method that can be used to improve both prediction accuracy and interpretation. This approach can estimate the regression coefficients for every feature and successively shrink them to avoid inflation of the estimated coefficients, resulting in superior predictive performance and irrelevant features. LASSO was conducted to choose the optimized subset of features to construct the final model.

**FIGURE 2:**
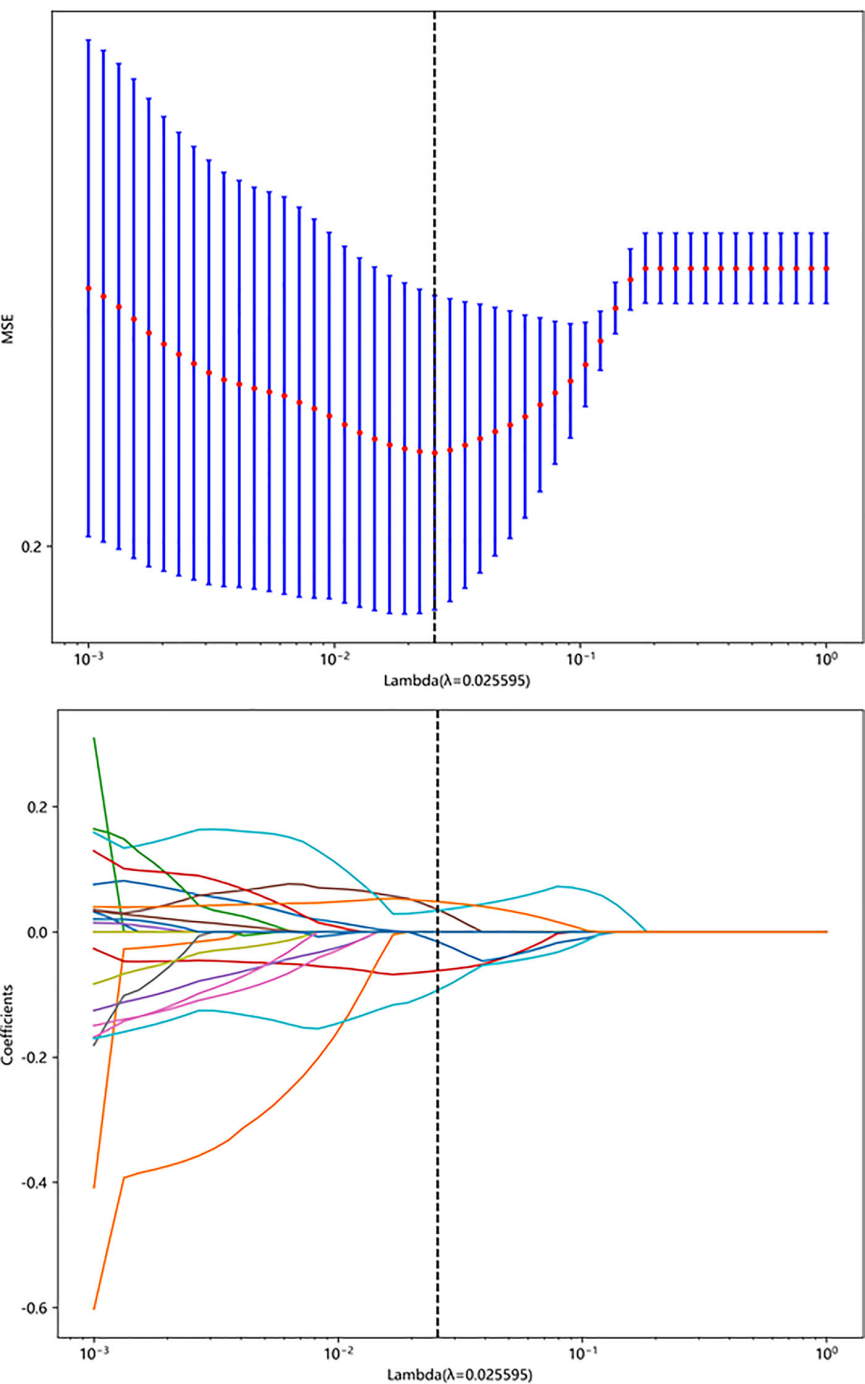
Texture feature selection using the least absolute shrinkage and the histogram of the Rad‐score based on the selected features. The optimal λ value of 0.026 was selected.

LASSO was first used to tune the regularization parameter λ using 10‐fold cross‐validation, and the optimized *λ* was corresponded to the minimal model bias. Then, the number of features was determined with the optimized *λ*, the most predictive subset of features was chosen and the corresponding coefficients were evaluated (Figure 2). The radiomics signature (Radscore) was calculated by summing the selected features weighted by their coefficients.

### 2.6 Prediction build and diagnostic validation

**FIGURE 3:**
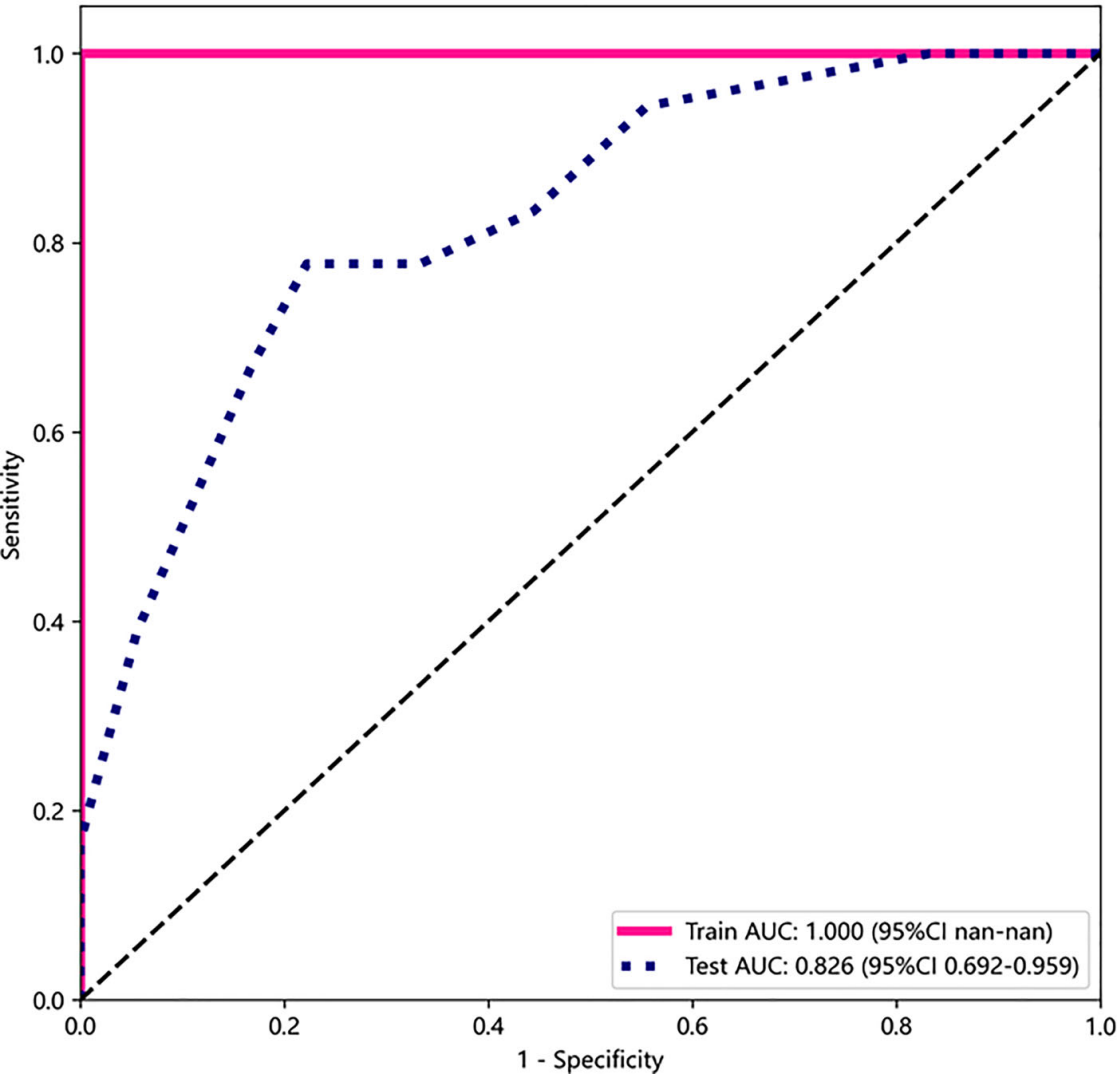
ROC curves of the radiomics mode in training set and test set. AUC, the area under the ROC curve (AUC); ROC, receiver operating characteristic curve.

A nomogram was formulated to predict MCI based on the results of LASSO regression. The sensitivity, specificity, accuracy, positive‐predictive value, and negative‐predictive value were obtained from the models. The predictive performance of the radiomics model was assessed using receiver operating characteristic (ROC) curve analysis (Figure 3).

### 2.7 Clinical usefulness

**FIGURE 4:**
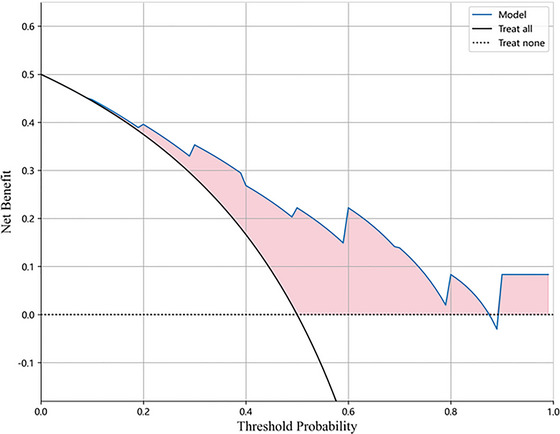
Decision curve analysis for the aMCI. The x‐axis represents the threshold probability, and the y‐axis represents the net benefit. aMCI, amnestic mild cognitive impairment.

Decision curve analysis (DCA) was applied to justify the clinical usefulness of this study. The prediction model provided clinical consequences regarding the choice of the threshold probability from which the net benefit could be derived. DCA was performed to estimate the clinical value of the radiomics nomogram by quantifying the net benefits based on the threshold probabilities. Net benefit is defined as the ratio of true positives minus the fraction of false positives weighted by the relative harm of false‐positive and false‐negative results. This threshold probability, *Pt*, is where the expected benefit of treatment equals the expected benefit of avoiding treatment (Figure 4).

### 2.8 Calibration curves of the radiomics model

**FIGURE 5:**
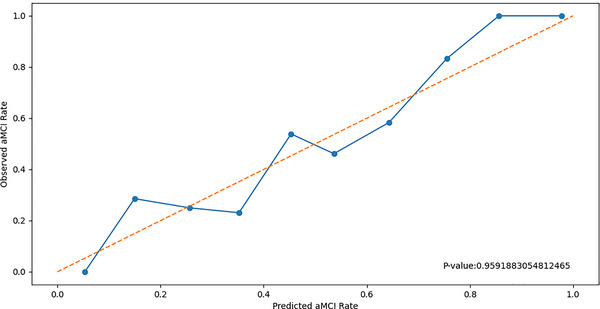
Calibration curves of the radiomics model. The y‐axis represents the incidence rate of aMCI. The x‐axis represents the predicted risk of aMCI. The diagonal dotted line represents the prediction of an ideal model. The blue solid line represents the performance of the radiomics model. A closer fit to the diagonal dotted line indicates a more accurate prediction. aMCI, amnestic mild cognitive impairment.

Calibration curves were plotted to evaluate the calibration of the radiomics model. Additionally, the Hosmer–Lemeshow test was conducted. Calibration curves show how well each model is calibrated by comparing the predicted risks of aMCI to the observed outcomes of aMCI (Figure 5).

### 2.9 Statistical analysis

Statistical analyses were conducted with python software (Version 3.7; https://www.python.org).

Variables for the normal control group and aMCI group were tested for normal distribution. In a two‐tailed analysis, a *p*‐value less than .05 was defined as statistically significant. We used an independent sample *t*‐test to evaluate the measurement variables and used the chi‐square test to compare the categorical variables. The reproducibility of intra‐observer and inter‐observer segmentation was assessed by inter‐class correlation coefficient and(ICC). The predictive performance of the models was evaluated using ROC analysis.

## 3 RESULTS

### 3.1 Baseline characteristics of the patients

Fourteen subjects were excluded from the study because they could not cooperate for a normal examination; 19 subjects were excluded because their image quality did not meet the diagnostic requirements. Finally, 126 patients with aMCI and 176 normal controls (NC) were included in this study. Table 1 shows the statistical analysis results for the demographic. There were no significant differences in age, gender, and education between the two groups (*p* > .05; Table 1).

**TABLE 1:** Clinical characteristics.

Sample size	aMCI (*N* = 91)	NC (*N* = 91)	*t*‐value	*p*‐value
Male (n,%)	69:57	92, 52.27%	2.43	0.18^a^
Age (years, mean ± SD)	56.750 ± 8.608	54.450 ± 7.34	−1.937	.054^b^
BMI (m/kg^2^,mean ± SD)	24.063 ± 1.419	23.663 ± 1.379	−1.928	.055^b^
Education (years, mean ± SD)	10.940 ± 4.088	9.984 ± 3.444	1.706	.090^b^
MoCA	25.740 ± 3.116	20.010 ± 3.264	12.103	<.01^b^
Systolic pressure (mmHg, mean ± SD)	134.680 ± 18.782	136.840 ± 17.896	−0.792	.429^b^
Diastolic pressure (mmHg, mean ± SD)	86.000 ± 12.130	83.650 ± 11.448	1.345	.180^b^

Abbreviations: aMCI, amnestic mild cognitive impairment; BMI, body mass index; MoCA, Montreal Cognitive Assessment; NC, normal controls.

^a^ *p*‐value for sex distribution obtained by the independent‐sample *t*‐test;

^b^ *p*‐value obtained using the independent‐sample t‐test.

### 3.2 Feature selection and LASSO logistic regression results

A total of 108 radiomic features were extracted from 3D‐T1WI imaging. Among the 108 extracted features, an ICC to or higher than 0.84 were included. To identify the relevant predictors, all explanatory features extracted from MR images of the training set were included in LASSO logistic regression. Features with regression coefficients of zero were eliminated. In the final feature selection with the LASSO method, 58 radiomic features were included from the T1‐weighted, including log‐sigma (*n* = 18), original (*n* = 8), and wavelet (*n* = 32) features, respectively (Figure 2).

### 3.3 Diagnostic performance of the radiomics model

The diagnostic performance of the radiomics model to distinguish aMCI from normal subjects was evaluated using ROC analysis in both the training set and the test set. In the training set, the area under the ROC curve (AUC) was 1.00, and the accuracy, sensitivity, and specificity were 100%, 98%, and 100%, respectively. In the testing set, AUC was 0.82 (95% Confidence Interval(CI):0.69–0.95), and the accuracy, sensitivity, and specificity were 69%, 92%, and 55%, respectively (Table 2, Figure 3).

**TABLE 2:** Model representation.

Task	Accuracy	AUC	95% CI	Sensitivity	Specificity	PPV	NPV
Training set	1.00	1.00	NA	0.98	1.00	1.00	1.00
Test set	0.69	0.82	0.69–0.95	0.92	0.55	0.65	0.76

Abbreviations: AUC, area under the receiver operating characteristic curve; NPV, negative‐predictive value; PPV, positive‐predictive value ;NA, not applicable.

### 3.4 Clinical usefulness

The DCA of the radiomics model is shown in Figure 4. The DCA shows that the radiomics model achieves a high net benefit. When the threshold probability is < 0.07 and > 0.83, the use of a radiomics nomogram based on the T1WI sequence might offer a higher net benefit.

### 3.5 Calibration curves of the radiomics model

The y‐axis represents the incidence rate of aMCI. The x‐axis represents the predicted risk of aMCI. The diagonal dotted line represents the prediction of an ideal model. The blue solid line represents the performance of the radiomics model. A closer fit to the diagonal dotted line indicates a more accurate prediction. The Hosmer–Lemeshow test resulted in a *p*‐value of .959. There was no statistically significant difference between the predicted values and the true values (Figure 5).

## 4 DISCUSSION

Early diagnosis of MCI and timely clinical intervention may slow down further decline of cognitive function and progression to AD (Livingston et al., 2020). Compared to subjective clinical characteristics, brain imaging biomarkers may provide more quantitative measures for suspected MCI (D. Yang & Hong, 2021). In this study, we developed a radiologic model and validated that MRI‐based radiologic features can effectively predict aMCI.

Radiomics is a promising technology that emerged in recent years, which improves the diagnosis and prediction efficiency of diseases by extracting a large number of radiomic features of target organs (H. Yang et al., 2022). In the present study, we extracted classes of quantitative radiomics features from 3D‐T1WI frontal white matter to evaluate the frontal lobe micro‐environment changes of patients with aMCI. ROC analysis indicates that radiomic features might effectively predict MCI patients. Log‐sigma features represent the variation in pixel intensities within a local neighborhood. High log‐sigma values indicate large variations and texture. The term LoG denotes the Laplacian of Gaussian filter, which serves as an edge enhancement filter. It accentuates regions in the image where there are changes in gray levels. The sigma value determines the extent to which texture features are enhanced. A smaller sigma value prioritizes the enhancement of fine textures, which correspond to gray‐level changes that occur over a short distance. Conversely, a larger sigma value emphasizes coarser textures, associated with gray‐level changes that occur over a larger distance (van Griethuysen et al., 2017). The employment of wavelet transform allows for the examination of multiple scales, facilitating the enhancement of subtle contrast disparities between lesions and normal tissues. In the realm of medical image diagnostics, log‐sigma, original, and wavelet features are typically utilized in conjunction with other feature types, such as first order, gray level co‐occurrence matrix, gray level dependence matrix, gray level run length matrix, and so forth rather than being employed individually (Speckter et al., 2022). Textural features possess widespread utility in the field of imaging and pathology, proving particularly valuable in the realm of diagnosis. As an example, they are employed in evaluating hippocampal dentate gyrus following cortical injury (Pantic et al., 2020). Furthermore, texture feature analysis serves as an effective approach for distinguishing between various types of amyloid plaques based on their morphology, as amyloid plaques are significant indicators of AD (Zaletel et al., 2021). Although research exploring the correlation between radiomics features and pathology is still in its preliminary stages, it holds promising potential.

Accumulating evidence suggests different imaging abnormalities related to AD follow a consistent trajectory during the pathogenesis of the disease, and that the first changes can be detected years before the disease manifests clinically (Pievani et al., 2011; Teipel et al., 2015). These findings have inspired clinical interest in using specific imaging markers to predict dementia development in patients at risk. MCI is a neurodegenerative disease caused by endogenous neuronal factors, including τ and amyloid pathology (Yu et al., 2017). As the key enzymatic player in the amyloidogenic processing of the amyloid precursor protein, β‐secretase activity increases have been observed in the temporal lobes and the frontal lobes of AD patients (Fukumoto et al., 2002). This may reflect the white matter contraction, interrupted connectivity, or loss of projection fibers in these cortical areas (Chalmers et al., 2005). The frontal‐subcortical circuits mean that projections are progressively focused onto a smaller number of neurons as they pass from cortical to subcortical structures, but circuit segregation is maintained (Cummings, 1993). Previous studies report AD and aMCI show parallel disruption of default mode network (DMN) resting state function and connectivity, as well as inter‐areal white matter pathway abnormalities (Alderson et al., 2017). In addition, medial frontal deactivation disrupts the DMN, decreasing AD patient activation and reactivity (Petrella et al., 2007; Rombouts et al., 2009). The DMN is engaged in attending to environmental stimuli, planning future behaviors, self‐awareness, and conscious processes (Petrella et al., 2007). A longitudinal study suggested that baseline hypoperfusion of the right middle frontal cortex predicts MCI progression to AD (Chao et al., 2010). Furthermore, the frontal cortex is not an isolated neuron unit and regional interaction and functional connection are inevitable during information processing. For example, the medial prefrontal cortex primarily interacts with the dorsal anterior cingulate cortex, especially in ambiguous situations requiring subjective interpretation (Nakao et al., 2010). These studies validated frontal‐subcortical circuit damage and the destruction of white matter integrity, which is related to the frontal lobe executive function and processing speed of aMCI and AD, and may be an important reason for the decline of cognitive function of aMCI and AD (Chalmers et al., 2005). Tau protein plays a key role in the pathological process of AD and is the main component of the characteristic neurofibrillary tangles that disrupt neuronal function (Sanford, 2017). Disease progression causes regional neuronal loss and consequent brain atrophy (Cummings & Cole, 2002). The atrophy of the hippocampus is involved the earliest and its atrophy significantly increased rates in aMCI and AD, with frontal lobe involvement occurring later in the disease (Scahill et al., 2002). However, many postmortem studies have implicated the entorhinal cortex and the transentorhinal region as early sites of degeneration in MCI and AD (Kordower et al., 2001). Therefore, the atrophy of brain tissue should be studied more clearly in aMCI and AD.

Recently, automatic‐aided diagnoses of AD and MCI based on ROI techniques, voxel‐based morphometry and volumetric analysis, have gained increasing popularity (Chen et al., 2011; Liu et al., 2017). In recent decades, machine learning and deep learning have also achieved great success in the fields of medical image analysis, particularly in assisting brain disease diagnosis (Cao et al., 2020). With algorithm evolution, model efficacy identifying aMCI and AD has steadily improved. Folego et al. analyzed MRI images using convolutional neural networks (CNNs), achieving 0.97 classification accuracy distinguishing AD from NC (Folego et al., 2020). Basaia et al. also applied CNNs to 3D T1‐weighted images and achieved high levels of accuracy in all the classifications in AD and MCI, with the highest rates achieved in the AD versus HC classification tests using both the ADNI dataset only (99%) and the combined ADNI + non‐ADNI dataset (98%; Basaia et al., 2019). While deep learning algorithms efficiently detect disease, their black‐box nature means few studies elucidate underlying pathological features, limiting clinical interpretability (Zhu et al., 2021). As brain atrophy tends to occur locally, only select regions demonstrate marked MRI structural changes that strongly correlate with pathology, whereas other regions offer little useful information for differential diagnosis (Zhu et al., 2021). ROI approaches employing 3D T1 imaging reveal 3D texture features that can predict AD and aMCI progression. According to Kovalev et al., 3D texture features capture more spatial information with greater sensitivity and specificity than 2D techniques, though less accurately than deep learning (Kovalev et al., 2001). Our radiomics model in the testing set yielded an AUC of 0.82 (95% CI:0.69–0.95), and the accuracy, sensitivity, and specificity were 69%, 92%, and 55%, respectively. In a prospective population‐based study of dementia, the AUC for hippocampal volume was found to be 0.724 (Achterberg et al., 2014). Feng et al. demonstrated the validity of potential hippocampal radiomic biomarkers for the diagnosis of aMCI. The AUC for the right hippocampus was 0.76, while for the left hippocampus, it was 0.79 (Q. Feng et al., 2019). However, Feng et al. demonstrated that the AUC for predicting aMCI based on hippocampal radiomics was 0.63 (F. Feng et al., 2018). The disparity in performance could stem from variations in feature selection criteria and model choices, resulting in a significant deviation in ROC results. This discrepancy may also be attributed to the utilization of different preprocessing methods, or the absence of a standardized preprocessing approach, prior to the extraction of radiomic features from the imaging data (Um et al., 2019). Compared with previous studies, the feature extraction method based on frontal white matter may better explain aMCI identification.

In clinical settings, as a conventional MR sequence, 3D‐T1 imaging is widely used in routine brain examination. Owing to the performing results of neuropsychological scales are unstable and require a high degree of patient cooperation (Guo et al., 2021), radiomic features could provide a more objective and convenient aMCI biomarker to help clinicians render prompt diagnoses.

However, there are several limitations in the present study. First, this study was a single‐center study with small sample sizes, and multi‐center with large samples external validation is further needed. Second, this study is based on the Chinese community population, the results may not generalize to other ethnic groups due to ethnic differences. Although the study only examined the frontal lobe, available evidence indicates that the frontal lobe pathology occurs during aMCI and AD progression. Finally, the study lacked an AD patient group.

## 5 CONCLUSION

In this study, we identified significant alterations in radiomic features related to aMCI within the frontal lobe white matter. These alterations demonstrated the ability to discriminate aMCI from NC with a relatively high diagnostic efficacy as evidenced by an AUC of 0.82. The calibration curve of the radiomics model indicated that the predictions align well with an ideal model. Furthermore, DCA illustrated a high net benefit achieved by the radiomics model. In conclusion, the radiomics features of the 3D‐T1 frontal lobe white matter may serve as a surrogate imaging marker to aid clinicians in screening for aMCI.

## AUTHOR CONTRIBUTIONS

Wei Zheng and Xiqi Zhu designed the study. Wei Zheng, Xiaoyan Qin, Ronghua Mu, and Xiaoyan Qin conducted the MRI data processing and statistical analyses. Wei Zheng, Ronghua Mu, Xin Li, JL, PY and Fuzhen Liu contributed to data collection and analyses. Wei Zheng and Ronghua Mu wrote the paper. Xiqi Zhu and Wei Zheng critically revised the manuscript. All authors approved the final draft.

## CONFLICT OF INTEREST STATEMENT

The authors declare that the research was conducted in the absence of any commercial or financial relationships that could be construed as a potential conflict of interest.

### PEER REVIEW

The peer review history for this article is available at https://publons.com/publon/10.1002/brb3.3222.

## Data Availability

The raw data supporting the conclusions of this article will be made available by the authors, without undue reservation.
